# Esophageal Variceal Ligation Monotherapy versus Combined Ligation and Sclerotherapy for the Treatment of Esophageal Varices

**DOI:** 10.1155/2021/8856048

**Published:** 2021-03-29

**Authors:** Jianbo Wang, Shenghui Chen, Yehia M. Naga, Junwei Liu, Mugen Dai, Shangwen Yang, Liangjing Wang, Bin Ye

**Affiliations:** ^1^Department of Gastroenterology, The Second Affiliated Hospital, College of Medicine, Zhejiang University, Hangzhou 310003, Zhejiang Province, China; ^2^Department of Gastroenterology, Lishui Hospital of Zhejiang University, Lishui Municipal Central Hospital, The Fifth Affiliated Hospital of Wenzhou Medical University, Lishui 32300, Zhejiang Province, China; ^3^Department of Gastroenterology, the First Affiliated Hospital, College of Medicine, Zhejiang University, Hangzhou 310003, Zhejiang Province, China; ^4^Division of Dig Ive Diseases, Department of Medicine, University of Mississippi Medical Center, Jackson, MS, USA

## Abstract

Currently, endoscopic variceal ligation (EVL) monotherapy is the standard therapy for managing esophageal variceal hemorrhage. Patients generally need several sessions of endoscopy to achieve optimal variceal ablation, and the varices can recur afterward. Endoscopic injection sclerotherapy (EIS) is an older technique, associated with certain complications. This study aimed to evaluate the clinical efficacy of EVL alone versus combined EVL and EIS in the treatment of esophageal varices. This retrospective study included 84 patients, of which 40 patients were treated with EVL monotherapy and 44 patients were treated with combined EVL + EIS. The main outcomes were rebleeding rates, recurrence at six months, number of treatment sessions, length of hospital stay, cost of hospitalization, and procedural complications. At six months, the rebleeding rate and recurrence were significantly lower in the EVL + EIS group compared to the EVL group (2.3% versus 15.0%; and 9.1% versus 27.5%, respectively). The number of treatment sessions, length of hospital stay, and cost of hospitalization were significantly lower in the EVL + EIS group compared to those in the EVL group (2.3 ± 0.6 versus 3.2 ± 0.8 times; 14.5 ± 3.4 versus 23.5 ± 5.9 days; and 23918.6 ± 4220.4 versus 26165.2 ± 4765.1 renminbi, respectively). Chest pain was significantly lower in the EVL + EIS group compared to that in the EVL group (15.9% versus 45.0%). There were no statistically significant differences in the presence of fever or esophageal stricture in both groups. In conclusion, combined EVL + EIS showed less rebleeding rates and recurrence at six months and less chest pain and was more cost effective compared to EVL alone in the treatment of gastroesophageal varices.

## 1. Introduction

Esophageal variceal bleeding (EVB) is a common complication of hepatic cirrhosis and is associated with significant morbidity and mortality [1, 2]. Half of the patients with cirrhosis will develop varices, with the annual incidence of EVB ranging from 5 to 15% and a 6-week mortality of 20% [3]. The increase of EVB events is associated with consequent medical expenses and high mortality, which has become a health burden worldwide.

At present, there are two main methods for the treatment of EVB, including endoscopic esophageal variceal ligation (EVL) and endoscopic injection sclerotherapy (EIS). EVL mainly depends on mechanical blockade of variceal flow to stop the bleeding and is the standard and preferred treatment modality. Despite the simple operation and the efficacy of recent hemostasis of EVL, higher recurrence rate after treatment remains a major concern [4, 5]. EIS is an older technique and can effectively eliminate varicose veins [1, 6]. Though EIS can achieve more complete hemostasis, it is inevitably associated with higher incidence of complications such as postoperative bleeding, chest pain, dysphagia, and esophageal stricture.

Recent studies have reported that EVL combined with EIS might be more efficient for the treatment of EVB [7–11], with lower recurrence rate and postoperative adverse events. However, most of the sclerotherapy injection points in these procedures are located between the gastroesophageal junction and the ligature ring, which is difficult in actual operation. Due to its limited clinical application, the safety and effectiveness of the combination treatment of EVL and EIS remain unclear.

Based on our previous clinical practice, we hypothesized that combination EVL + EIS approach can be potentially more efficient in variceal ablation and can result in a lower recurrence rate. We aimed to evaluate EVL alone versus combined EVL and EIS in the treatment of esophageal varices.

## 2. Patients and Methods

### 2.1. Patients and Data Collection

Patients with hepatic cirrhosis and EVB who were admitted to the Lishui Municipal Central Hospital, China, between January 2015 and June 2018, were retrospectively included in the study. The inclusion criteria were as follows: (1) patients with cirrhosis complicated by EVB. (2) Age between 20 and 80 years. (3) Endoscopic treatment within 48 hours of presentation. The exclusion criteria were as follows: (1) any history of current cancer or debilitating disease. (2) Other causes of upper gastrointestinal bleeding such as peptic ulcer disease. (3) Prior endoscopic treatment for esophageal varices. (4) Prior surgical treatment for esophageal varices such as transjugular intrahepatic portosystemic shunt (TIPS) or splenectomy. (5) Patients on propranolol or other nonselective beta blockers.

A total of 84 patients were included in this study. The patients were divided into two groups, 40 patients were treated with EVL alone and 44 patients were treated with combined EVL and EIS. In the EVL group, all patients underwent endoscopy within 48 hours from presentation, after fluid resuscitation and octreotide administration.

### 2.2. Ethics Statement

The study was conducted in accordance with the provisions of the 1975 Declaration of Helsinki and with the approval of the Ethics Committee of Lishui Municipal Central Hospital.

### 2.3. Materials and Endoscopic Treatment

EVL was performed using Six Shooter^®^ endoscopic banding device (Cook Medical, Winston-Salem, NC, USA). Variceal ligation was performed beginning at the most distal discernible variceal column and then proceeding to the next proximal varix. Each varix was ligated at 1–2 points, with ≤6 bands for each session.

**Figure 1:**
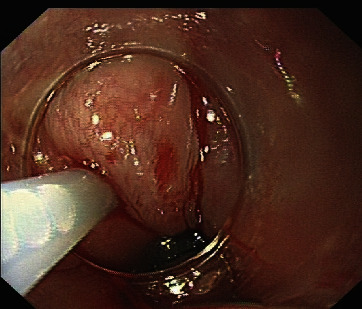
Sclerosant was injected into each varicose vein, approximately 2-3 cm above the ligation ring.

In the group of patients who received combined EVL and EIS, we chose the close possible location near the gastroesophageal junction. Each vein was ligated with one band, and excess air was pushed into the upper-middle section of the esophagus; directly after placing the needle biopsy channel. Each injection was 2–5 mL (maximum volume ≤ 20 mL) of sclerosant agent, which was injected in the varix during ligation, 2–3 cm proximal to the placed band (Figure 1). We used lauromacrogol (Tianyu Pharmaceutical, Shanxi, China) as the sclerosant.

### 2.4. Follow-up and Outcome Assessment

Follow-up endoscopic exams were conducted between 14 and 21 days, and repeated treatments were administered until complete eradication of the esophageal varices was achieved. During each follow-up endoscopic exam between 14 and 21 days, the sclerosant volume used for each session was between 2 and 10 mL. The follow-up endoscopic exams were conducted until complete eradication of the esophageal varices.

The main outcomes measured for the two groups were rebleeding rate, recurrence at six months, number of treatment sessions, length of hospital stay, cost of hospitalization, and complications from treatment.

### 2.5. Statistical Analysis

SPSS 23.0 was used for statistical analysis. Data were expressed as mean ± standard deviation (mean ± SD), and a *t* test was used for comparison. If the data did not conform to a normal distribution, a *U* test was adopted. A *p* value < 0.05 was considered statistically significant.

## 3. Results

### 3.1. Patient Characteristics and Clinical Outcomes

On the index endoscopic examination, the number of esophageal variceal columns in the EVL group was 2.2 ± 1.0, while in the EVL + EIS group was 2.2 ± 0.9. The varices in the EVL group were classified as esophageal varices without gastric varices in 5.0% of the patients (2/40), while 80% (32/40) were classified as GOV1 and 15% (6/40) as GOV2. In the EVL + EIS group, 6.8% (3/44) were esophageal varices without gastric varices, 72.3% were GOV1, and 20.5% (9/44) were GOV2. Further endoscopic manifesations are outlined in Table 1. The average number of endoscopic sessions for the EVL + EIS group was 2.3 ± 0.6, which was significantly less than that in the EVL group (3.2 ± 0.7). The total length of hospitalization in the EVL + EIS group was 14.5 ± 3.4 days, which was significantly less than the 23.50 ± 5.94 days in the EVL group. The total cost in the EVL + EIS group was 23918.6 ± 4220.4 renminbi, which was less than that of the EVL group at 26165.2 ± 4765.1 renminbi. There was a significant difference between the two groups (Table 2).

### 3.2. Clinical Assessment and Complications

Rebleeding occurred in one patient (2.3%) in the EVL + EIS group and six patients (15.0%) in the EVL group. Chest pain occurred in seven patients (15.9%) in the EVL + EIS group and 18 patients (45.0%) in the EVL group. Eight patients in the EVL + EIS group (18.2%) reported dysphagia, which was also reported in 10 patients in the EVL group (25.0%). Fever was observed in three patients (7.5%) in the EVL + EIS group and in six patients (13.6%) in the EVL group. No patient had empyema, perforation, or embolization. In the EVL + EIS group, four patients (9.0%) had recurrence at six months, while in the EVL group, 11 patients (27.5%) had recurrence at six months.

Chest pain was significantly lower in the EVL + EIS group compared to the EVL group (*n* = 7, 15.9% versus *n* = 18, 45.0%, *p*=0.004, respectively), while there were no statistically significant differences in the presence of fever or esophageal stricture in both groups (*n* = 4, 9.1% versus *n* = 4, 10.0%, *p* = 1; *n* = 6, 13.6% versus *n* = 3, 7.5%, *p*=0.58) (Table 3).

## 4. Discussion

EVL is the preferred treatment for esophageal varices. Its efficacy is comparable to that of sclerotherapy, but with less procedural bleeding, chest pain, dysphagia, esophageal stenosis, and other complications [7, 12]. Compared with EIS, EVL can eradicate esophageal varices in fewer sessions but has a higher recurrence rate mainly because EVL cannot ligate the collateral vein and deep vein. A sclerosant can chemically obliterate these veins. The longer the exposure, the better is the effect [7]. Rebleeding after treatment with EVL is often associated with a premature drop of the placed band, and the probability of the band falling off during the ligation of bulky varices is higher, which is one of the reasons why hospitals rarely use EVL. EVL + EIS can effectively prevent the band from falling off after bleeding.

There are many studies on the treatment of esophageal varices with band ligation and sclerotherapy. Tajiri et al. described injection sclerotherapy into esophageal varices and then ligating them [8]. Harras et al. performed EVL at 5–10 cm from the gastroesophageal junction and then injected the sclerosant in the varices near the ligation area, 2–3 cm from the gastroesophageal junction [9]. Mansour et al. [7], Saeed et al. [10], and Lo et al. [11] injected the sclerosant at the ligation point after EVL. However, in most of these studies, the sclerosant was injected at a point in space between the gastroesophageal junction and the deployed band and at the time of the ligation method. In our study, we used the closest possible location near the gastroesophageal junction. Each vein was ligated with one band, and excess air was pushed into the upper-middle section of the esophagus; directly after placing the needle biopsy channel, 2–5 ml (≤20 ml) of sclerosant agent was injected in the varix during ligation, 2–3 cm from the upper part of the band. Most vessels are located in the gastroesophageal junction, but our observation from gastric coronary vein angiography showed that many veins directly enter the esophagus more proximally in the upper-middle esophagus. Distal esophageal varices disappear in many patients after EVL, but the upper section of veins remains prominent, indicating that the method of injecting the sclerosant at a point between the gastroesophageal junction and band can prevent recurrence, which has been confirmed by Mansour [7]. Therefore, our method allows the sclerosing agent to remain in the vein for a longer time after the ligation band blocks part of the blood flow so that it exerts more chemical effect on the venous endothelium. In addition, the sclerosing agent can act on numerous veins in a wide range, starting from approximately 3–6 cm above the gastroesophageal junction, and all the veins above this point can be chemically affected by the sclerosing agent. We observed during the follow-up that the esophageal varices were visible and firm under the transparent cap, but this phenomenon usually subsided after 3–6 months with signs of bleeding or obvious blood flow after needle injections.

In the present study, the site of sclerosant was also a striking feature. Clinically, we found that both the oral and anal sides are effective for blocking the blood supply route of varices. We adopted the oral side as the site of sclerosant (Figure 1). The reasons were as follows: first, we performed the EVL and EIS in the same operation, and choosing oral side as injection location is clinically easier and safer. Second, after performing EVL, the blood flow of varicose veins was obviously blocked. Then, we performed EIS and pulled out the needles and found that the bleeding volume of the oral side was significantly less than the anal side.

Whether EVL and EIS should be performed on the same day remains controversial in previous reports [13, 14]. In this study, for the EVL + EIS group, we chose to perform the EVL and EIS on the same day for the first therapy, and subsequently, we only performed EIS until complete eradication of the esophageal varices was achieved. The reasons were as follows: first, performing EVL and EIS on the same day reduced the endoscopic operation times (2.3 ± 0.6 vs. 3.2 ± 0.8 times) and also decreased the length of hospital stay (14.5 ± 3.4 vs. 23.5 ± 5.9 days) and total cost (23918.6 ± 4220.4 versus 26165.2 ± 4765.1 renminbi). Second, performing EVL followed by EIS could also reduce the risk of bleeding after ligation of thick varicose veins.

At the six months follow-up, four patients (9.1%) in the EVL group and 11 patients in the EVL + EIS group (27.5%) had recurrence of esophageal varices. In the EVL group, the patients who relapsed were treated with sclerotherapy again, and later required TIPS. In the EVL + EIS group, band ligation was performed again without requiring TIPS. Only one patient (2.3%) in the EVL + EIS group experienced bleeding after start of endoscopic treatment, which occurred on the 16th day after the index banding session. In the EVL group, six patients (15.0%) presented with postoperative bleeding, all cases occurred within 5–12 days after the index ligation and involved bleeding due to premature falling of the band. There were four cases of esophageal stenosis in both groups after endoscopic treatment, none of which required dilation. The patients in the EVL + EIS group experienced remission in about 2–3 months, while the patients in the EVL group generally experienced remission in 15 days to 2 months. The dysphagia sensation in the EVL + EIS group appeared to result from a relatively longer stenosis in the distal esophagus, while in the EVL group, it was mainly caused by the postligation scar, which resolved quickly.

In summary, combined variceal ligation and sclerotherapy had less rebleeding rates and recurrence at six months and less chest pain and was more cost effective compared to endoscopic variceal ligation alone in the treatment of gastroesophageal varices. Randomized controlled studies are needed for better evaluation of these observations.

## Figures and Tables

**Table 1:** General data, Child grade, clinical manifesations, and initial endoscopic manifesations of patients in the two groups.

	EIS + EVL group (*n* = 44)	EVL group (*n* = 40)	*p* value
Age (years)	53.9 ± 9.7	54.5 ± 8.8	0.79
Gender			0.45
Male	35	29	
Female	9	11	
HBV	35	24	0.50
Initial endoscopic manifesations			
Number of bulky veins	2.2 ± 1.0	2.2 ± 0.9	0.97
Thrombus head	32	26	0.44
Ascites	17	19	0.41
Hepatic encephalopathy	1	2	0.93
Increased bilirubin	16	16	0.73
Child, grade			0.14
A	7	10	
B	33	22	
C	4	8	
Type of varices			0.36
Only the esophagus	3	2	
GOV1	32	32	
GOV2	9	6	

**Table 2:** Comparison of treatment times, total hospital stay, and total costs between the two groups.

Items	EIS + EVL group (*n* = 44)	EVL group (*n* = 40)	*p* value
Treatment times (times)	2.3 ± 0.6	3.2 ± 0.8	<0.001
Total hospital stay (days)	14.5 ± 3.4	23.5 ± 5.9	<0.001
Total costs (renminbi)	23918.6 ± 4220.4	26165.2 ± 4765.1	0.044

Values are given as mean ± SD.

**Table 3:** Complications in the two groups.

Complications	EIS + EVL group (*n* = 44)	EVL group (*n* = 40)	*p* value
Rebleeding, *n* (%)	1 (2.3)	6 (15.0)	0.087
Retrosternal pain, *n* (%)	7 (15.9)	18 (45.0)	0.004
Difficulty in swallowing, *n* (%)	8 (18.2)	10 (25.0)	0.45
Esophageal stricture, *n* (%)	4 (9.1)	4 (10.0)	1
Fever, *n* (%)	6 (13.6)	3 (7.5)	0.58
Recurrence, *n* (%)	4 (9.1)	11 (27.5)	0.028

## Data Availability

The data used to support the study are not freely available concerning the privacy of the patients.

## Abbreviations:

EVL: Endoscopic variceal ligation
EIS: Endoscopic injection sclerotherapy
EVB: Esophageal variceal bleeding
TIPS: Transjugular intrahepatic portosystemic shunt.

## Supplementary Materials

Supplementary Materials STROBE Statement—checklist of items that should be included in reports of observational studies.
